# Palmitic acid differently modulates extracellular vesicles and cellular fatty acid composition of SGBS adipocytes without impairing their insulin signaling

**DOI:** 10.3389/fendo.2025.1699831

**Published:** 2025-11-25

**Authors:** Domenico Sergi, Sharon Angelini, Fabiola Castaldo, Marco Beccaria, Carlo Bellinghieri, Flavio Franchina, Alice Omenetto, Gabriella Stifani, Sefora Del Mastro, Stefania Merighi, Stefania Gessi, Ravin Jugdaohsingh, Carlo Cervellati, Martin Wabitsch, Luca Maria Neri, Guillaume Bidault, Antonio Vidal-Puig, Juana Maria Sanz, Angelina Passaro

**Affiliations:** 1 Department of Translational Medicine, University of Ferrara, Ferrara, Italy; 2 Department of Chemical, Pharmaceutical, and Agricultural Sciences, University of Ferrara, Ferrara, Italy; 3 Biomineral Research Group, Department of Veterinary Medicine, University of Cambridge, Cambridge, United Kingdom; 4 German Center for Child and Adolescent Health (DZKJ), Partner Site Ulm, Department of Paediatrics and Adolescent Medicine, Division of Paediatric Endocrinology and Diabetes, Ulm University Medical Center Ulm, Ulm, Germany; 5 Institute of Metabolic Science, University of Cambridge, Cambridge, United Kingdom

**Keywords:** extracellular vesicles, adipocytes, palmitic acid, fatty acids, insulin resistance

## Abstract

**Scope:**

Adipocyte-derived extracellular vesicle (EV) lipid cargo reflects the obese metabolic state. Nevertheless, it is currently unknown whether the adipocyte-derived EV lipid profile is influenced by saturated fatty acid overload. This study investigated if palmitic acid (PA) affected human Simpson–Golabi–Behmel syndrome (SGBS) adipocyte insulin sensitivity and the repercussions on the EV fatty acid cargo secreted by these cells.

**Methods:**

Adipocytes were treated with 500 or 1,000 μM of PA, and cytotoxicity was assessed using the lactate dehydrogenase assay. Thereafter, cells were treated with 1,000 μM of PA for 48 h, followed by the assessment of triglyceride accumulation and adipokine expression. Insulin signaling, NF-κβ activation, and stearoyl-CoA desaturase-1 (SCD) abundance were assessed by Western blot. EVs were isolated via ultracentrifugation, and the intracellular as well as the EV fatty acid profile was characterized using gas chromatography coupled to a flame ionization detector.

**Results:**

Neither 500 nor 1,000 μM of PA did not elicited a cytotoxic effect on SGBS adipocytes. PA promoted adipocyte hypertrophy without hampering insulin signaling or triggering the activation of NF-κβ. However, PA increased intracellular and EV SFA content and raised intracellular oleic acid (OA) levels in parallel with the upregulation of SCD, while it decreased OA content in EVs.

**Conclusions:**

Active lipid sorting within EVs may be an additional mechanism underpinning intercellular communication by which adipocytes inform other cells about their metabolic status. However, further studies are warranted to evaluate the effects of EV lipid cargo on recipient cells.

## 1 Introduction

Obesity is a global health concern, having reached epidemic proportions worldwide (1). The magnitude of this global health crisis is exemplified by the close association between obesity and a plethora of diseases ranging from cancer to cardiometabolic aberrations (2–5). In particular, obesity hampers cardiometabolic health by fostering the development of type 2 diabetes mellitus (T2D) (3), metabolic syndrome (6), and cardiovascular disease (5). Adipose tissue hypertrophic expansion and dysfunction are at the nexus between obesity and its dire cardiometabolic aberrations (7, 8). In keeping with this, increased lipid spillover and deregulated adipokine secretion from dysfunctional adipose tissue fuel lipotoxicity and low-grade chronic inflammation, which are both pivotal in promoting insulin resistance (9–12). The latter, in turn, is a crucial discriminant in shaping cardiometabolic health (13, 14).

The adipose tissue, besides its role in buffering and releasing energy, which is stored in adipocytes in the form of triglycerides, is an endocrine organ (15). As such, it secretes a wide array of mediators, termed adipokines, which modulate systemic energy metabolism and insulin sensitivity (16, 17). Adipose tissue-derived metabolic cues are also conveyed, in the form of proteins, lipids, and microRNAs (miRNAs), by extracellular vesicles (EVs) (18). EVs are generated via the inward cell membrane budding and are incorporated into intracellular multivesicular bodies of endosomes as intraluminal vesicles. Multivesicular bodies fuse with the plasma membrane, leading to the secretion of intraluminal vesicles from the parent cells as exosomes (19). EVs, apart from representing a significant component of adipose tissue secretome (20), are also emerging as a contributor to obesity-related metabolic dysfunctions (21, 22), given their ability to modulate energy metabolism, insulin sensitivity, and inflammatory response, among others (23). Accordingly, the concentration of plasma EVs was reported to be higher in obese individuals compared to their lean counterparts and to positively correlate with homeostatic model assessment for insulin resistance (HOMA-IR) (24, 25). Additionally, the adipose tissue has been proposed as the primary source of circulating EVs (26) with plasma adipose tissue-derived EVs increasing in individuals with obesity and type 2 diabetes (27) as well as in obese mice (28). The ability of EVs to contribute to obesity-associated metabolic complications has been mainly attributed to their protein (29–31) and miRNA (26, 32, 33) cargo. However, EVs secreted from the adipose tissue are also a source of bioactive lipids, which can be shuttled to other cells and tissues in order to shape their metabolism (34, 35). The potential of EV lipid cargo to affect metabolic health is further corroborated by the fact that obesity has also been shown to shape the lipid cargo of EVs secreted by the adipose tissue (36). In keeping with this, the amount of lipid species containing stearic and arachidonic acid has been increased in the EVs secreted from the visceral adipose tissue of obese mice (36). Therefore, a shift in the lipid cargo of adipose tissue EVs may contribute to the metabolic aberrations driven by adipose tissue dysfunction secondary to obesity. Moreover, palmitic acid (PA, C16:0), known to exert obesogenic effects (16, 37) and to impair cardiometabolic health (38), not only increases in the circulation of individuals with T2DM (39) but also has been shown to affect the fatty acid composition of EVs secreted from either murine or human adipocyte cell models (25, 40). However, to the best of our knowledge, it has not been investigated whether the ability of PA to modulate the fatty acid (FA) cargo of EVs secreted from adipocytes is paralleled by an impairment of adipocyte response to insulin signal, which leads to the onset of insulin resistance. Thus, the aim of this study is twofold: first, to elucidate whether PA overload impairs human Simpson–Golabi–Behmel syndrome (SGBS) adipocyte insulin sensitivity, and second, to evaluate if this is coupled with a shift in their EV fatty acid cargo.

## 2 Experimental section

### 2.1 Cell culture and fatty acid treatment

SGBS human adipocytes were gifted by Professor Martin Wabitsch (University Hospital of Ulm, Germany). Functionally, SGBS adipocytes show a gene expression pattern comparable to primary preadipocytes and mature human adipocytes and provide a unique model system to investigate cell metabolism and the influence of nutrients or drugs on human fat physiology (41). SGBS human preadipocytes were cultured in Dulbecco’s modified Eagle’s medium/nutrient mixture F-12 (DMEM/F12) supplemented with 10% fetal bovine serum (FBS), pantothenic acid (17 μM), biotin (33 μM), penicillin (100 U/mL), and streptomycin (100 μg/mL), until 80%–90% confluence was reached. To induce their differentiation into mature adipocytes, cells were incubated for the following 14 days, using two different differentiation media: Quick-media was added for the first 4 days [DMEM low glucose (5.5 mM), pantothenic acid (17 μM), biotin (33 μM), penicillin (100 U/mL), streptomycin (100 μg/mL), transferrin (0,01 mg/mL), insulin (20 nM), cortisol (100 nM), triiodothyronine (T3) (0.2 nM), dexamethasone (DEXA) (25 nM), isobutylmethylxanthine (IBMX) (250 μM), and rosiglitazone (2 μM)]. For the remaining 10 days, cells were exposed to 3FC-media: DMEM low glucose (5.5 mM), pantothenic acid (17 μM), biotin (33 μM), penicillin (100 U/mL), streptomycin (100 μg/mL), transferrin (0,01 mg/mL), insulin (20 nM), cortisol (100 nM), and triiodothyronine (T3) (0.2 nM) (41). Differentiation was performed in the presence of 5.5 mM glucose to prevent nutrient overload, which, instead, was administered in the form of PA post-differentiation as described below. Cells were maintained in a humidified incubator at 37°C in a 5% CO_2_ atmosphere. Fully differentiated adipocytes were treated with either low-endotoxin 250 μM bovine serum albumin (BSA) or 500 or 1,000 μM of PA for 48 h in DMEM low glucose (5.5 mM), and then samples were collected for downstream analysis. Depending on the experimental procedure, cells were grown in 6- or 24-well plates. The former were used for the assessment of gene expression and protein abundance by Western blot, whereas the latter were for the experiments aimed at tracking fluorescently labeled PA.

### 2.2 Conjugation of palmitic acid to bovine albumin serum

PA was conjugated to BSA as described previously (42, 43). Briefly, PA was dissolved in 0.1 M of NaOH in a block heater at 70°C to yield a final concentration of 20 mM. In parallel, BSA was dissolved in serum and penicillin–streptomycin-free DMEM low glucose at a final concentration of 0.5 mM. Once PA was fully dissolved and the solution was free of visible precipitates, the PA-NaOH solution was diluted 10-fold in the low-endotoxin, fatty acid-free BSA–DMEM solution to obtain a 4:1 molar ratio (PA 2 mM:BSA 0.5 mM). The control BSA was obtained following the same protocol described above. However, the BSA solution was not mixed with PA but with 0.1 M of NaOH only, which was consequently diluted 10-fold. The solution was then vortexed for 10 s and incubated at 55°C for 10 min in order to allow the PA–BSA conjugation to occur. Finally, the PA–BSA mix was cooled to room temperature, filter-sterilized, and stored at −20°C until use.

### 2.3 LDH cytotoxicity assay

Adipocytes were treated with PA at different concentrations, 500 and 1,000 μM, for 48 h. LDH cytotoxicity assay was performed on the conditioned media following the manufacturer’s instructions. Briefly, 100 μL of conditioned media was plated in a 96-well plate, followed by 100 μL of reaction mixture, and incubated for 30 min at room temperature. Then, 50 μL of stop solution was added, and absorbance was recorded at 500 nm using a Tecan Infinite^®^ 200 PRO spectrophotometer (Tecan Trading AG, Switzerland).

### 2.4 NBD-palmitic acid labeling assay

Adipocytes were treated for 48 h with 1,000 μM of PA in the presence of 10 μM of fluorescently labeled 7-nitrobenz-2-oxa-1,3-diazol-4-yl (NBD)-PA (Avanti Research^™^—a Croda brand, Alabama, USA). After treatment, cells were washed twice with prewarmed PBS and then fixed for 15 min with 4% paraformaldehyde at room temperature, followed by two washes with PBS. Finally, coverslips were mounted onto glass slides using Fluoroshield™ with DAPI (Sigma-Aldrich, Milano, Italy) to counterstain the nuclei. Images were acquired using the fluorescent microscope Nikon Eclipse 5i equipped with a cooled charge-coupled device (CCD) camera Nikon DS-Qi1 and analyzed with NIS Elements v 5.11 software.

### 2.5 Triglyceride quantification assay

Following the exposure to PA or BSA, adipocytes were washed with PBS and lysed with RIPA buffer containing ethylenediaminetetraacetic acid (EDTA) (30 mM), NaCl (150 mM), Nonidet-40 (1% v/v), deoxycholic acid (0.5%), sodium dodecyl sulfate (SDS) (0.1%), and tris(hydroxymethyl)aminomethane (TRIS) (50 mM). Intracellular triglyceride relative quantification was assessed within the cell lysates using the Roche Diagnostics colorimetric assay kit (Indianapolis, USA) following the manufacturer’s instructions. Briefly, 10 μL of cell lysate was mixed with 250 μL of a triglyceride reaction mix in a 96-well plate, which was then incubated at 37°C for 10 min. Finally, absorbance was read at 500 nm using the Tecan Infinite^®^ 200 PRO spectrophotometer (Tecan Trading AG, Switzerland).

### 2.6 Measurement of the diameter of lipid droplets

The size of the intracellular lipid droplets was measured using ImageJ software. Briefly, the diameters of lipid droplets, categorized as small, medium, and large, were measured in 5 random cells per 10 different fields (1 field = 1 image), for each treatment. The analysis was performed by three staff members to obtain three data pools per treatment, which were then averaged.

### 2.7 Western blotting

Following 48 h of treatment with BSA or PA, cells were incubated with 100 nM of insulin for 15 min or directly lysed with RIPA buffer in the presence of a protease and phosphatase inhibitor cocktail (Sigma-Aldrich).

Protein concentration was quantified using the BCA Protein Assay Kit (Sigma-Aldrich), and 10 μg of proteins were resolved using a 7.5% polyacrylamide gel. Proteins were then transferred onto a PVDF membrane using the Trans-Blot Turbo Transfer System (Bio-Rad), and non-specific binding sites were blocked by incubating the membrane with 5% non-fat dried milk. After blocking, the membranes were probed overnight with primary antibodies: anti-pAkt (Ser473) (Cell Signaling, Leiden, The Netherlands), anti-pIRS (Tyr612) (Sigma-Aldrich), anti-IκBα (Sigma-Aldrich), anti-stearoyl-CoA desaturase-1 (SCD) (Sigma-Aldrich), anti-Alix (Cohesion Biosciences, London, UK), and anti-β-actin (Sigma-Aldrich), followed by three 5-min washes with PBS containing 0.1% Tween-20 (PBS-T). Consecutively, the membrane was probed with HRP-linked secondary antibody (1:10,000) for 2 h at room temperature and then washed with PBS-T as described above. Protein bands were revealed using Clarity Western ECL substrate (Bio-Rad) and captured using Invitrogen iBright Imagers (Thermo Fisher Scientific, USA), while densitometry analysis was performed using iBright Analysis Software (Thermo Fisher Scientific).

### 2.8 RNA extraction, cDNA synthesis, and RT-qPCR gene expression assessment

After treatments, cells were directly lysed using the Aurum Total RNA Lysis Solution (Bio-Rad Laboratories, USA) and stored at −80°C until use. RNA was extracted using the Aurum Total RNA Mini Kit DNA-free (Bio-Rad), after which 500 ng was retro-transcribed into cDNA using 5× PrimeScript RT Master Mix (TaKaRa, Japan) according to the manufacturer’s instructions. The expression of interleukin-6 (IL-6), interleukin-1β (IL-1β), tumor necrosis factor-α (TNF-α), monocyte chemoattractant protein-1 (MCP-1), leptin, and adiponectin was assessed by the StepOne Plus Real-Time PCR system and using the TB Green Premix Ex Taq (Tli RNaseH Plus) (TaKaRa) master mix. The PCR reaction conditions were set as follows: 1 cycle at 95 °C for 30 s, followed by 40 cycles at 95 °C for 5 s and 30 s of the annealing phase, whose temperature was primer set-specific as detailed in **Table 1**. Data were normalized to *rRNA 18S* and analyzed using the comparative ΔCt method.

**Table 1:** Gene expression, primer sequences, and annealing temperature.

Gene	Sequence 5′–3′	T annealing (°C)
Leptin (forward)	TCCACCCCATCCTGACCTTAT	60
Leptin (reverse)	GGTTCTCCAGGTCGTTGGAT	
Adiponectin (forward)	GTGAGAAAGGAGATCCAGGTCTT	59
Adiponectin (reverse)	GGCACCTTCTCCAGGTTCTC	
PCG1α (forward)	AGGCTGGCAGTGTGCTG	60
PCG1α (reverse)	TGGTCACTGCACCACTTGAG	
IL-6 (forward)	GGAGACTTGCCTGGTGAAAA	60
IL-6 (reverse)	GTCAGGGGTGGTTATTGCAT	
IL-1β (forward)	ACAGATGAAGTGCTCCTTCCA	59
IL-1β (reverse)	GTCGGAGATTCGTAGCTGGAT	
TNFα (forward)	AAGCACACTGGTTTCCACACT	60
TNFα (reverse)	TGGGTCCCTGCATATCCGTT	
MCP-1 (forward)	AGCCACCTTCATTCCCCAAG	60
MCP-1 (reverse)	CTCCTTGGCCACAATGGTCT	
18S (forward)	GTAACCCGTTGAACCCCATT	60
18S (reverse)	CCATCCAATCGGTAGTAGCG	

### 2.9 Isolation of extracellular vesicles

Conditioned media were collected from cells treated with BSA or PA and centrifuged at 1,500×*g* for 20 min to remove cell debris (44). Supernatants from four biological replicates of BSA- or PA-treated cells were then pooled in a 5.1-mL ultracentrifugation tube and subjected to ultracentrifugation at 100,000×*g* for 1 h at 4°C using the TLA-100.4 fixed-angle rotor (Beckman, USA) and the Beckman Optima™ Series TL ultracentrifuge (Beckman, USA). EV pellets were collected and washed with sterile PBS and recentrifuged at 100,000×*g* for 1 h at 4°C. EVs were collected in 350 μL of sterile PBS and stored at −80°C until further analysis.

### 2.10 Nanoparticle tracking analysis

The size and concentration of the particles contained in the EV samples were assessed using the NanoSight NS500 device (Malvern Panalytical, Malvern, UK). Briefly, the EV suspension was diluted 1 in 500, and the sample was then injected. Data analysis was performed using the NTA 3.4 Build 3.4.003 software (Malvern Panalytical).

### 2.11 Transmission electron microscopy analysis

EV pellet suspension was deposited on Formvar^®^-coated copper grids and fixed for 20 min with 1% glutaraldehyde and 2% paraformaldehyde in phosphate buffer adjusted to pH 7.4. After six washes with Milli-Q water, EVs were negatively stained with 0.5% uranyl acetate for 10 min. Grids were rinsed briefly with Milli-Q water and left to air dry overnight. Grids were then observed with the transmission electron microscope Talos L120-C G2 (Thermo Fisher Scientific), operated at 120 keV.

### 2.12 Lipidomic analysis of intracellular and EV fatty acids

#### 2.12.1 Chemical, standard, and analytic reagents

*n*-Hexane (99%), methanol (≥99%), potassium hydroxide, n-alkanes (from *n*-C7 to *n*-C30), undecanoic acid (C11:0), tricosanoate methyl ester (Me.C23:0), and SUPELCO C37 mixture of fatty acid methyl esters (FAMEs) were obtained from Merck, Milano Italy.

#### 2.12.2 One-step derivatization

The lipidomic analysis was performed either on cell lysates and EV samples obtained from BSA or PA-treated cells (*n* = 4). To derivatize in one step both esterified and non-esterified fatty acids present in biological samples into FAMEs, a modification of the method developed by Liu and coworkers was used (45). Briefly, 50 μL of each biological sample was transferred into 2-mL screw-cap vials. To each sample, 10 μL of undecanoic acid (C11:0, 100 ppm) as an internal standard solution was added. Subsequently, 1 mL of 1% sulfuric acid (H_2_SO_4_) in anhydrous methanol was added, and the vials were incubated at 50°C overnight (>8 h) to induce fatty acid derivatization. After cooling to room temperature, 150 μL of Milli-Q water, 300 μL of n-hexane, and an additional 10 μL of tricosanoate methyl ester (Me.C23:0, 100 ppm) as a second internal standard were added to both cellular and EV-extracted lipid samples to verify the correct completion of the derivatization process. The samples were vortexed for 10 s, and the upper organic phase containing FAMEs was collected. The extracted organic phase was transferred to a new vial, ready for GC analysis (45).

#### 2.12.3 GC-FID parameters

Analyses of FAMEs were carried out on a GC system 8860 (Agilent Technologies, Milan, Italy) equipped with a non-polar J&W HP-5 GC column, 30 m × 0.32 mm i.d. × 0.25 μm film df (stationary phase: 5% phenyl-methylpolysiloxane and 95% dimethylpolysiloxane). One microliter was injected in split mode (split ratio 1:10), and the oven temperature program was from 60°C at 5°C/min to 300°C and from 20°C/min to 320°C. Injector temperatures were set at 280°C. A flame ionization detector (FID) was coupled to a GC instrument, with a temperature set at 350°C. Helium was used as carrier gas at a constant linear velocity of 27.081 cm/s and an initial head pressure of 7.8 psi. For FID conditions, the sampling frequency was 20 Hz. Data were processed using the Agilent OpenLab CDS (Agilent Technologies, Milan, Italy).

#### 2.12.4 Reliability of one-step FAME derivatization and GC-FID analysis

FAMEs of cell lysates and EV samples obtained from BSA- or PA-treated cells were analyzed in triplicate (*n* = 12). To monitor the entire analytical procedure, two internal standards, namely, undecanoid acid (C11:0) and tricosanoate methyl ester (Me.C23:0), were used. Undecanoic acid went under the same derivatization method applied for the samples, while Me.C23:0 was added before the GC injection to monitor the instrumental variation. **Supplementary Figure S2** reports the charter control of undecanoic C11:0 acid and Me.C23:0 internal standards. The area of the internal standards was under the warning limits (average value ± 2 * standard deviation) considering all the analyses (*n* = 12), highlighting the repeatability of the entire methodology. FAMEs were identified using standards and their linear retention index (LRI) in a range of ±10 under non-polar column elution (46).

### 2.13 Statistical analysis

Data were expressed as the mean ± SEM. Experiments were conducted at least twice to confirm the results. Samples, which underwent GC-FID characterization, represent a pool of four independent wells, and the statistical analysis was performed on three technical replicates. The difference between treatments was assessed by an unpaired Student’s *t*-test using GraphPad Prism 8 for Windows. A *p <*0.05 was considered statistically significant.

## 3 Results

### 3.1 Palmitic acid is readily incorporated into lipid droplets and increases intracellular triglyceride levels

**Figure 1:**
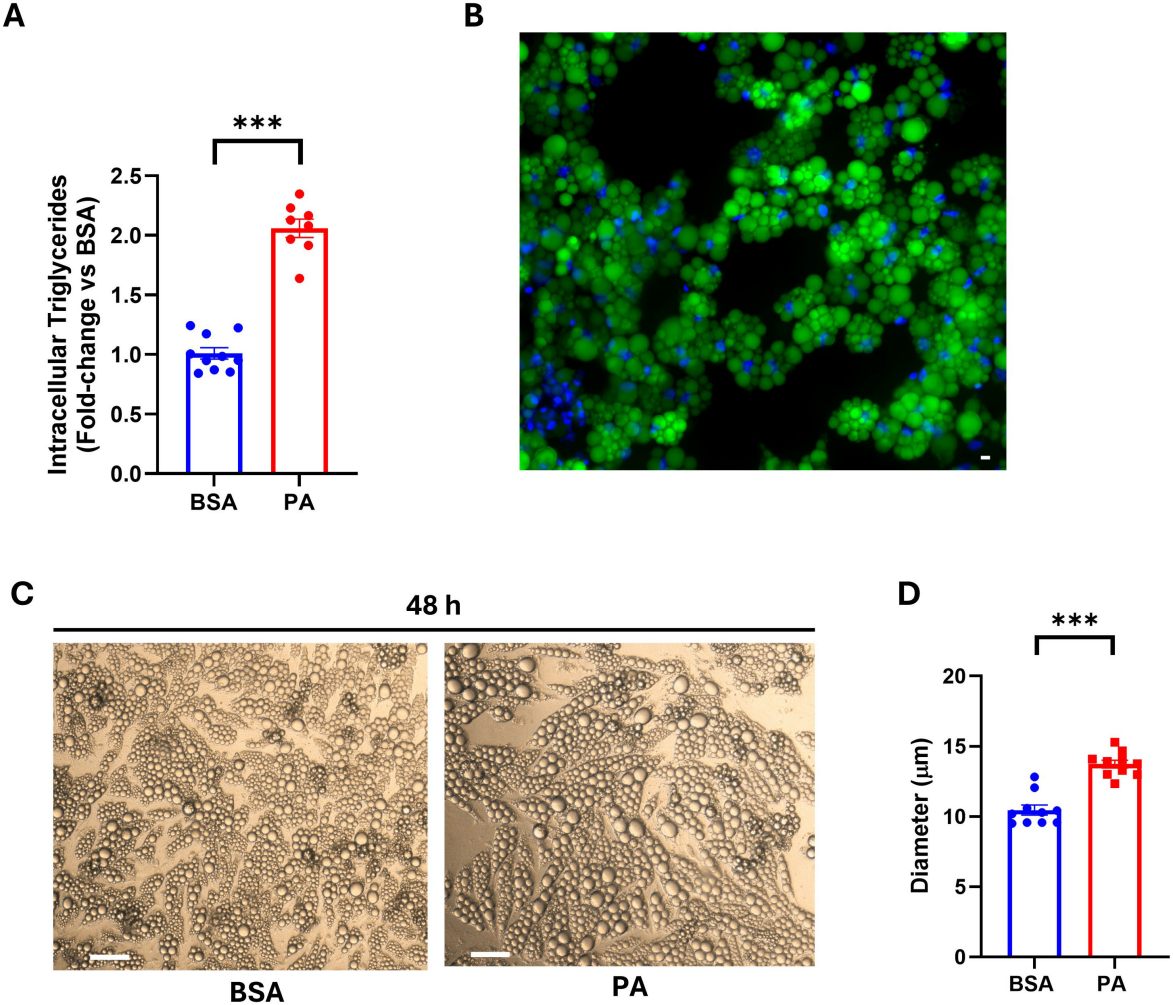
Intracellular triglyceride accumulation and SGBS adipocyte lipid droplet size following palmitic acid (PA) exposure. **(A)** Intracellular triglyceride quantification on cell lysate after 48 h of treatment with PA. **(B)** Lipid droplet localization of fluorescently labeled PA assessed using fluorescence microscopy. Scale bar = 50 μm. **(C)** Bright-field images of SGBS adipocytes after 48 h of treatment with bovine serum albumin (BSA) or PA. Scale bar = 100 μm. **(D)** Diameter of intracellular lipid droplets of BSA- vs. PA-treated cells. Intracellular triglyceride data are expressed as fold change of BSA-treated cells and represented as mean ± SEM. Columns report the mean of at least eight independent wells ± SEM, while lipid droplet size is reported as the mean of *n* = 10 measurements, for each condition as detailed in the materials and methods. ***p < 0.001.

Adipocytes are crucial in buffering excess energy by channeling it toward storage in the form of neutral lipids. Chronic energy overload, in turn, leads to adipocyte hypertrophy and dysfunction (47). Thus, once the PA concentration that did not elicit a cytotoxic effect was identified, we evaluated whether energy overload, in the form of this SFA, was able to increase intracellular triglycerides and if this fatty acid was incorporated into lipid droplets. PA, when used at 1,000 µM, did not elicit a cytotoxic effect (**Supplementary Figure S1**); therefore, all experimental procedures thereafter were conducted using this fatty acid concentration. However, this SFA successfully raised intracellular triglyceride compared to BSA-treated cells (**Figure 1A**). Additionally, the increase in NBD-palmitic acid-related fluorescence in lipid droplets (**Figure 1B**) demonstrates that, at least in part, exogenous PA was stored in adipocyte lipid droplets. These findings were further confirmed by the ability of PA to increase the size of adipocyte lipid droplets (**Figures 1C, D**).

### 3.2 Palmitic acid did not impair insulin signal transduction

**Figure 2:**
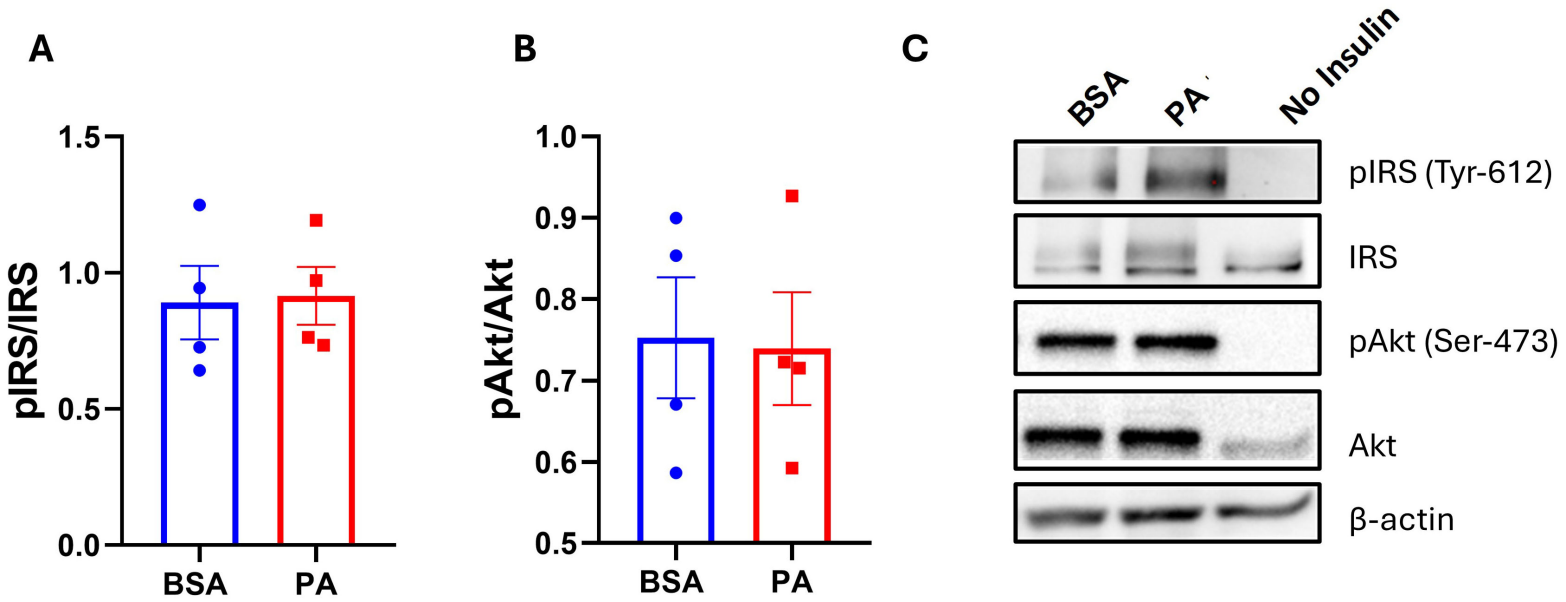
The effect of palmitic acid (PA) on insulin-induced phosphorylation of insulin receptor substrate (IRS-1) and protein kinase B (Akt). Densitometric analysis of **(A)** IRS-1 and **(B)** Akt/PKB normalized to their respective total proteins. **(C)** Representative Western blot of phosphorylated IRS-1 and Akt/PKB abundance. Data are represented as the mean of four independent wells ± SEM.

Energy overload is one of the key factors in promoting hypertrophy and dysfunction in adipocytes, which is characterized by reduced insulin sensitivity (47). Despite an increase in triglyceride accumulation, the 48-h treatment with PA failed to hamper the phosphorylation of both IRS-1 (Tyr612) (**Figures 2A, C**) and Akt/PKB (Ser473) (**Figures 2B, C**) in response to insulin stimulation.

### 3.3 Palmitic acid did not trigger the activation of the NF-kB pro-inflammatory pathway nor upregulate cytokine expression

**Figure 3:**
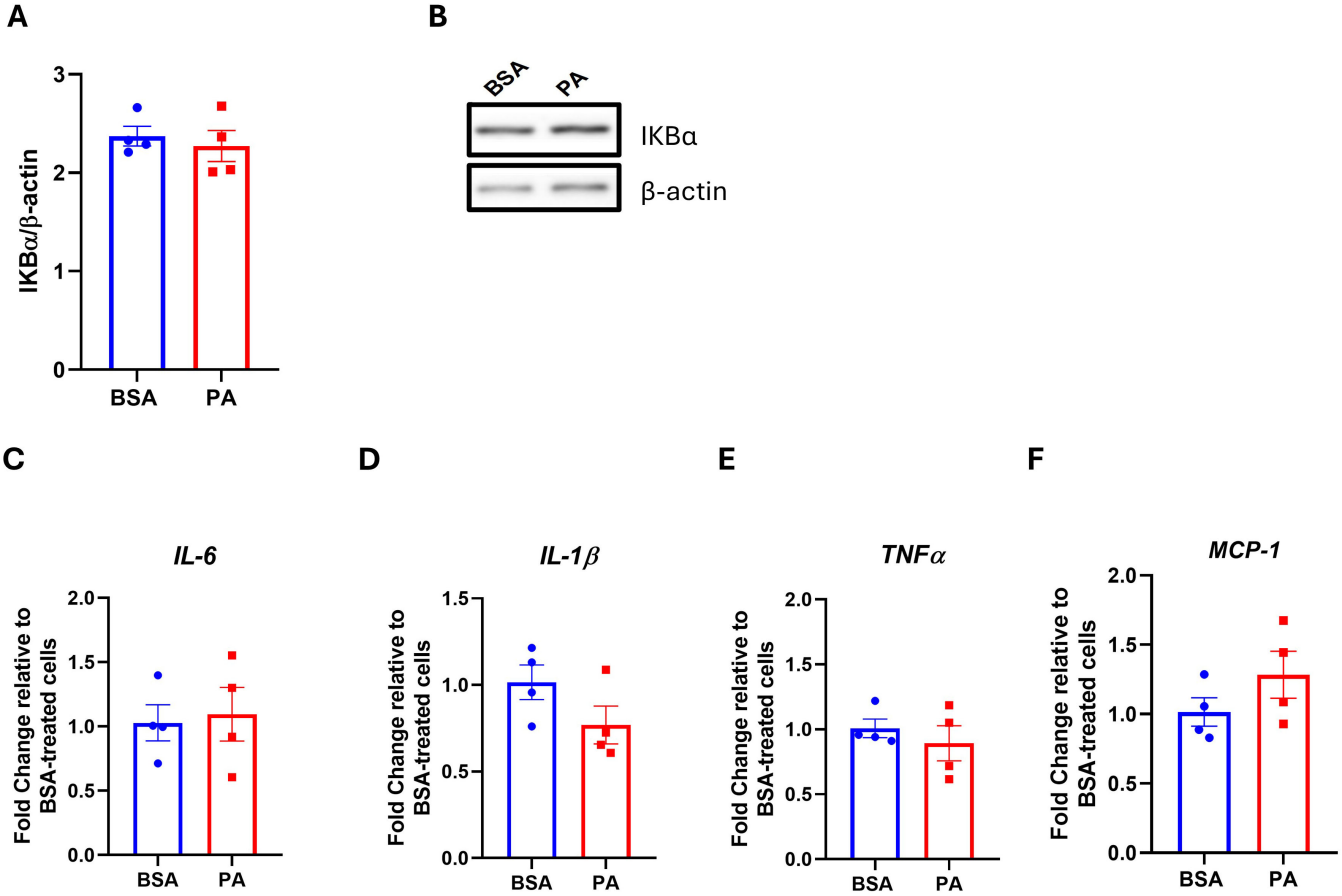
The effect of palmitic acid (PA) on the IKKB–NF-κB signaling pathway and the expression of pro-inflammatory mediators. Densitometric analysis of **(A)** the inhibitor of NF-κB (IκB-α) normalized to β-actin and **(B)** its representative Western blot. Gene expression analysis of the pro-inflammatory cytokines **(C)** interleukin-6 (IL-6), **(D)** interleukin-1β (IL-1β), and **(E)** tumor necrosis factor-α (TNFα) and **(F)** the chemokine monocyte chemoattractant protein-1 (MCP-1). Gene expression data are expressed as fold change compared to BSA-treated cells **(C–F)**. Data are reported as the mean of four independent wells ± SEM.

The activation of the NF-κβ pro-inflammatory pathway, as well as an increase in pro-inflammatory mediators, represents a key characteristic of adipocyte dysfunction and contributes to insulin resistance, particularly in response to SFA overload (48). Since PA did not hamper insulin-mediated phosphorylation of IRS-1 and Akt, it was next investigated whether this may relate to the inability of this SFA to activate pro-inflammatory responses in this model. In line with the preserved insulin signaling, PA did not activate the NF-κβ signaling pathway as demonstrated by the absence of reduction in the inhibitor of NF-κβ (IkB-α) relative to BSA (**Figures 3A, B**). In addition, PA did not affect the expression of the pro-inflammatory mediators *IL-6* (**Figure 3C**), *IL-1β* (**Figure 3D**), *TNF-α* (**Figure 3E**), and *MCP-1* (**Figure 3F**). Altogether, our results demonstrate that PA treatment over 48 h did not trigger a pro-inflammatory state in SGBS adipocytes.

### 3.4 Palmitic acid did not impact leptin to adiponectin expression level

**Figure 4:**
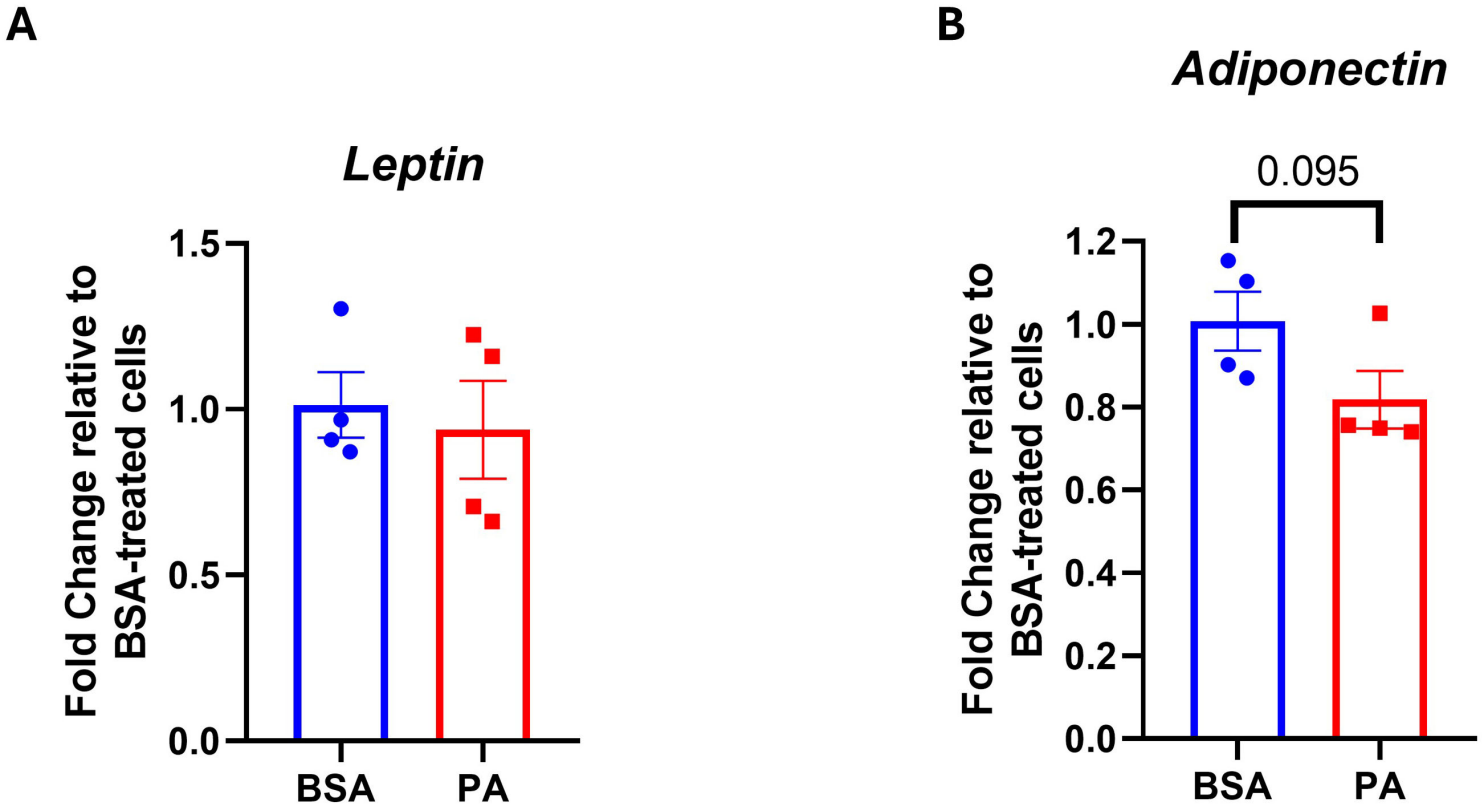
Adipokine gene expression in response to palmitic acid (PA). Gene expression analysis of **(A)** leptin and **(B)** adiponectin. Data were expressed as fold change compared to BSA-treated cells and represented as the mean ± SEM of four independent wells.

A decrease in the adiponectin–leptin ratio is indicative of adipocyte dysfunction. Therefore, we evaluated whether PA modulates the expression of adiponectin and leptin. While we did not observe any significant change in leptin expression (*p* > 0.05) (**Figure 4A**), PA tended to downregulate adiponectin expression (*p* = 0.095) (**Figure 4B**).

### 3.5 Palmitic acid influenced intracellular and EV fatty acid quality

**Figure 5:**
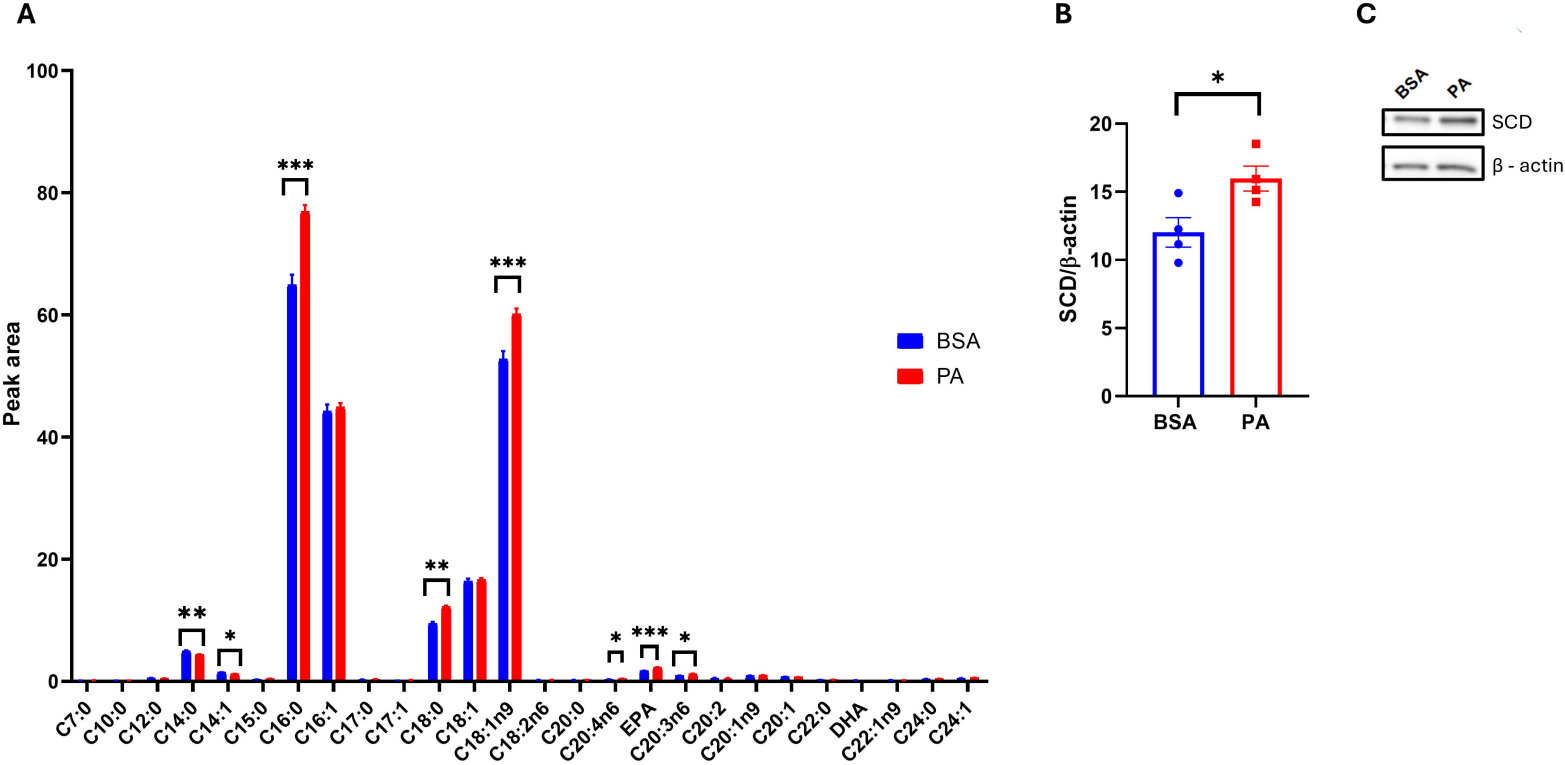
SGBS adipocyte fatty acid profile in response to palmitic acid (PA). **(A)** Intracellular fatty acid profile comparison between bovine serum albumin (BSA)- and PA-treated adipocytes. **(B)** Densitometric analysis of stearoyl-CoA 9-desaturase (SCD) normalized to β-actin and **(C)** its representative Western blot. Data are reported as the mean of four independent wells ± SEM. GC-FID data represent the mean ± SEM of the pool of four independent wells of BSA- and PA-treated cells, while statistical analysis was performed on three technical replicates. Western blot data are the mean ± SEM of four independent wells. *p < 0.05, **p < 0.01, ***p < 0.001.

While PA failed to promote adipocyte dysfunction, this may be disentangled from changes in adipocyte secretome and particularly their EV quantity and quality, notably regarding their fatty acid composition. To address this question, we first characterized the intracellular fatty acid composition of SGBS adipocytes after 48 h of exposure to PA. PA treatment increased intracellular levels of palmitic acid (C16:0, *p* < 0.001) and stearic acid (C18:0, *p* < 0.01), suggesting that a portion of the exogenous palmitate underwent elongation via ELOVL6 activity. In addition, PA significantly elevated the levels of the monounsaturated fatty acid oleic acid (C18:1n-9, *p* < 0.001; **Figure 5A**), while the levels of palmitoleic acid (C16:1n-7) remained unchanged. Notably, PA exposure also increased mRNA expression of the Δ9-desaturase SCD (*p* < 0.05; **Figures 5B, C**), suggesting that SGBS cells responded to the accumulation of SFAs by enhancing their desaturation capacity, a mechanism likely aimed at mitigating lipotoxic stress (49). Of note, the absence of increased C16:1n-7 and C18:1n-7 implies that, in SGBS cells, SCD preferentially targets stearic acid over palmitic acid.

Interestingly, PA treatment also reduced the levels of myristic acid (C14:0) and myristoleic acid (C14:1) (*p* < 0.001), while increasing several polyunsaturated fatty acids (PUFAs), particularly arachidonic acid (C20:4n-6), eicosapentaenoic acid (EPA), and dihomo-γ-linolenic acid (C20:3n-6) (*p* < 0.001; **Figure 5A**).

**Figure 6:**
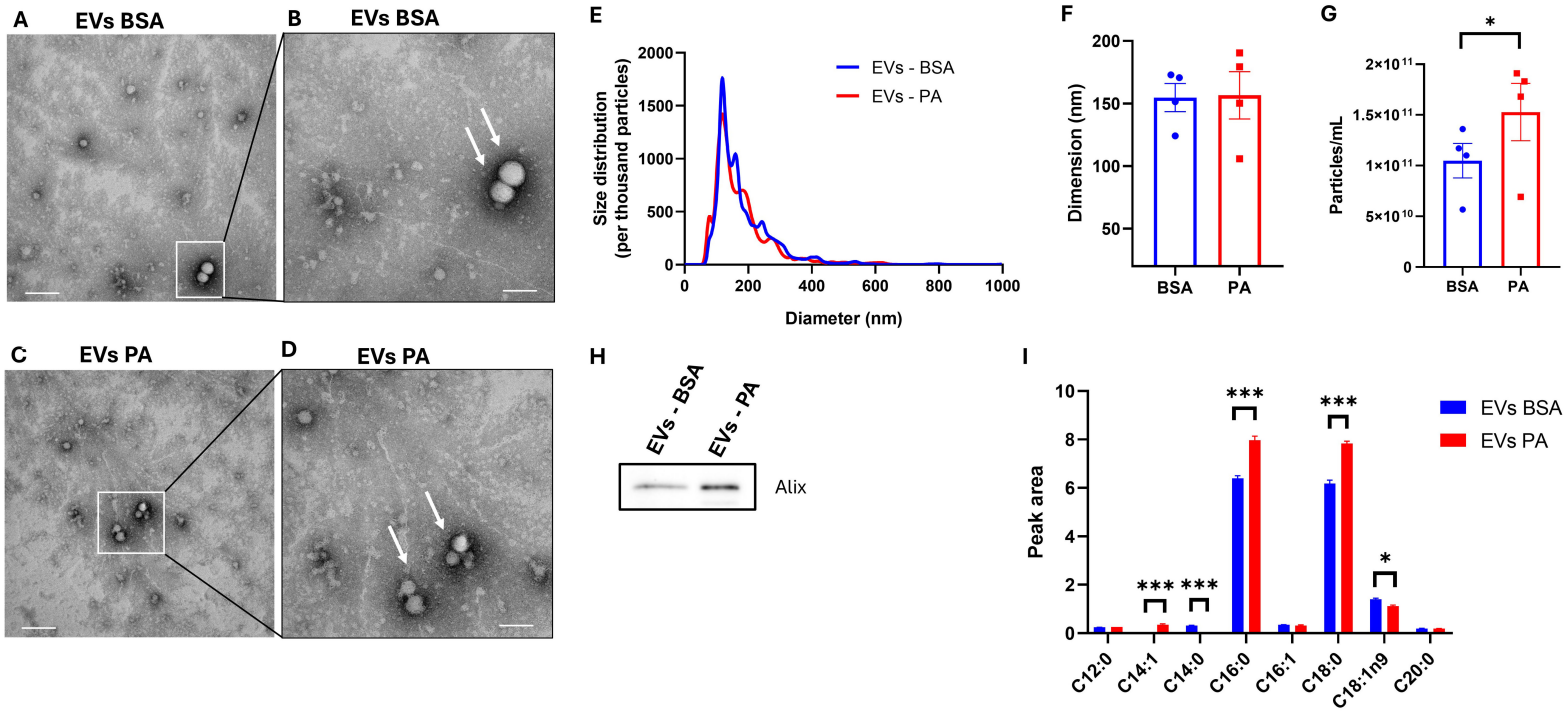
Extracellular vesicle (EV) fatty acid profile following exposure of SGBS adipocytes to palmitic acid (PA). **(A)** Image from transmission electron microscopy analysis representative of EVs isolated from the conditioned media of BSA at ×36,000 magnification (scale bar = 100 nm) and **(B)** at ×73,000 magnification (scale bar = 200 nm). **(C)** Image from transmission electron microscopy analysis representative of EVs from PA-treated cells at ×36,000 magnification (scale bar = 200 nm) and **(D)** at ×73,000 magnification (scale bar = 100 nm). **(E)** EV size distribution comparison between control and PA-treated cells normalized per thousand of particles. Particles size **(F)** and counts **(G)** obtained with NTA analysis. Data from NTA analysis are expressed as the mean ± SEM of *n* = 4 independent experiments. **(H)** EV fatty acid profile comparison between bovine serum albumin (BSA)- and PA-treated adipocytes. GC-FID EV data are reported as the mean ± SEM of the three technical replicates of a pool of four independent wells of BSA- and PA-treated cells. *p < 0.05, **p < 0.01, ***p < 0.001.

It was next investigated that, besides modulating the intracellular fatty acid profile, PA affected the fatty acid exported as part of EVs. First, transmission electron microscopy analysis confirmed the isolation of EVs from both control (**Figures 6A, B**) and treated cells (**Figures 6C, D**). EV isolation was further confirmed by identifying the presence of the exosomal marker Alix in EV secreted by BSA- and PA-treated cells (**Figure 6H**).

EV derived from PA-treated cells did not show ultrastructural morphological differences when compared to control EV (**Figures 6A–D**). Moreover, nanoparticle tracking analysis (NTA) showed comparable size distribution between the EVs released from both control and PA-treated cells (**Figure 6E**) with a particle mean size of approximately 150 nm (**Figure 6F**). However, although there were no changes in the size distribution and dimension of the particles, PA increased the number of particles released (**Figure 6G**).

GC-FID analysis revealed that the fatty acid composition of EVs is mainly characterized by SFA. Particularly, to the same extent as the intracellular compartment, 48 h of exposure to PA induced an increase in C16:0 and C18:0 (*p* < 0.001) (**Figure 6E**). However, there was an increase in the absolute amount of C14:1 (undetected in BSA-treated cells) and a decrease in the levels of C14:0 (undetected in PA-treated cells) and C18:1ω-9 (*p* < 0.05) (**Figure 6E**). Furthermore, SFA represented the most abundant fatty acids detected in the EVs with the relative abundance of C18:1 and C18:0 being inverted in the intracellular compartment (**Figure 5A**) compared to the EVs (**Figure 6E**).

## 4 Discussion

The data reported herein describe the ability of a 48-h PA overload to modulate the fatty acid cargo of EVs secreted by human SGBS adipocytes in parallel with the induction of their hypertrophy but without hampering insulin signaling or triggering the activation of pro-inflammatory pathways.

The adipose tissue is crucial for buffering energy excess, which is stored in the form of triglycerides with adipocyte lipid droplets (50). This physiological function appears preserved in SGBS adipocytes, which are able to channel some of the exogenous PA into lipid droplets. However, chronic nutrient overload, in the presence of defective adipose tissue expandability, promotes adipocyte hypertrophy and consequent dysfunction, leading to insulin resistance (51), as observed in 3T3-L1 murine adipocytes (52–54). PA did not impair insulin signaling in SGBS adipocytes despite inducing an increase in lipid droplet size and intracellular triglyceride accumulation. Additionally, while some studies reported that PA triggers inflammatory responses in murine adipocytes (55, 56), this was not corroborated in our study. This further confirms the ability of these cells to cope with a PA overload, at least for 48 h. Channeling of palmitate into neutral lipids could explain this effect, as previously observed *in vivo* (57, 58). This suggests that SGBS cells possess a unique metabolic adaptability that allows them to cope with SFA overload without initiating inflammatory pathways. However, this increase in energy storage within lipid droplets was potentially not sufficient to overwhelm adipocyte storage capacity and may explain the inability of PA to trigger inflammation and hamper insulin signaling. The discrepancy in terms of the ability of PA to hamper insulin signaling in previous studies (52–54, 59) but not in the present study may be attributed to differences in experimental conditions, such as the purity and percentage of BSA used and the glucose concentration in the culture medium. Also, in line with this, another report provided evidence that PA can impair insulin signaling and trigger inflammatory responses in SGBS adipocytes despite using a lower PA concentration compared to the present study (60). These discrepancies may be explained by the different protocols used for conjugating PA to BSA, with PA being dissolved in ethanol. Another potential difference may be in the type of BSA used, with the present study employing exclusively low-endotoxin BSA for all the experimental procedures. In keeping with this, standard fatty acid-free BSA, but not low-endotoxin BSA, has been reported to activate pro-inflammatory responses via the Toll-like receptor 4 (61). Additionally, exposing SGBS adipocytes to PA in the presence of higher glucose concentrations (17.5 mM in DMEM/F12) (60) or 4.5 g/L glucose (59) compared to those used in the present study (5.5 mM in low-glucose DMEM) may have exacerbated the lipotoxic effects of this long-chain saturated fatty acid (62). Finally, the differences in terms of the ability of PA to promote insulin resistance in adipocytes may be cell model-specific. In line with this, the present study investigated the effect of this SFA on human adipocytes, while other studies focused on murine cell models (52–54, 59) whose capacity to cope with a lipid overload may be lower compared to SGBS cells. Nonetheless, another study, despite reporting data on murine 3T3-L1 adipocytes, highlighted the inability of PA to alter lipolysis, trigger an inflammatory response, and downregulate adiponectin despite increasing intracellular lipid content (63).

In agreement with the above-discussed findings, PA did not promote adipocyte dysfunction as indicated by the inability of this SFA to hamper insulin signal transduction and trigger inflammatory responses. In spite of this, PA modulated the adipocyte as well as EV fatty acid composition, albeit to a different extent. Indeed, the PA challenge decreased the intracellular levels of myristic and myristoleic acids while increasing the levels of PA itself along with stearic and oleic acids. The increase in oleic acid is in agreement with the upregulation of SCD, which, in turn, is responsible for the synthesis of monounsaturated fatty acids, including oleic acid (64). Thus, PA may be intracellularly converted to stearic acid, which is then desaturated by SCD in order to synthesize oleic acid. This unsaturated fatty acid, in turn, may potentially prevent the metabolically detrimental effect of PA on SGBS adipocytes by exerting anti-inflammatory effects (65). Moreover, desaturation of saturated fatty acids by SCD may be necessary to channel exogenous SFA toward TAG and the lipid droplet (66) and enhance fat storage in adipocytes (67, 68). Hence, the upregulation of SCD may represent an adaptive mechanism able to prevent PA-induced lipotoxicity and metabolically detrimental effects (69), albeit until adipocyte critical storage capacity is reached. Furthermore, SGBS adipocytes treated with PA display higher levels of the polyunsaturated fatty acid dihomo-gamma linolenic acid, arachidonic acid, and EPA. Given that cells were cultured in serum-free conditions, an external source of PUFAs can be excluded. Therefore, these changes likely reflect selective retention of long-chain PUFAs, possibly due to impaired β-oxidation or stress-induced metabolic remodeling. Moreover, PA may lower EPA mobilization and metabolism, thereby increasing the intracellular levels. However, this hypothesis remains to be confirmed. Altogether, these findings indicate that SGBS cells actively remodel their lipid composition in response to palmitate exposure, likely to preserve lipid homeostasis, which is consistent with the observed maintenance of insulin sensitivity and the absence of an inflammatory response.

Regarding the lipid cargo of EVs secreted by SGBS adipocytes, this is mainly made up by the saturated fatty acids PA and stearic acid, whereas oleic acid, which was the second most abundant fatty acid in SGBS adipocyte lysate, was lower compared to both the aforementioned saturated fatty acids. This confirms previous findings, indicating that adipocytes selectively sort lipid into EVs (25, 36), which, therefore, may act as an additional adipocyte-derived cue to modulate systemic metabolic health. The ability of adipocytes to actively sort lipids within EVs is influenced by nutrient challenges (25, 40). In keeping with this, PA increased the proportion of saturated fatty acids, particularly PA and stearic acid, while lowering the oleic acid content of EVs secreted from SGBS adipocytes. This finding is partially in agreement with a previous study, which reported an increase in stearic and a decrease in oleic acid in the EVs secreted from SGBS adipocytes in response to a PA challenge (25). Nonetheless, while the data reported herein indicated that PA treatment also increased the level of this fatty acid within EVs, previously published data indicated that PA only induced intracellularly in SGBS adipocytes following PA treatment (25). This difference may be dependent on the experimental settings used in this study and in a previously published report, which differ in the duration of the exposure to PA. Additionally, as part of this study, PA was administered in serum-free DMEM, whereas it was previously supplemented to FBS-containing 3FC media (25). As proposed previously (25), the increase in saturated fatty acid secretion within EVs may represent an additional protective effect that adipocytes put in place in order to prevent the lipotoxic effect elicited by excess SFA. Nevertheless, despite lipid sorting being a tempting hypothesis, also supported by the fact that adipocyte-derived EVs stem from the central lipid droplet of adipocytes (34), EV lipid composition may be a reflection of the adipocyte plasma membrane. Indeed, the increase in stearic acid in the EV fatty acid fraction may indicate an enrichment in adipocyte plasma membrane lipids, as this saturated fatty acid, as opposed to oleic, is more readily incorporated into cellular phospholipid (70). Additionally, while overall SFAs are more abundant than monounsaturated fatty acids in EVs, PA induced an increase in both C16:0 and C18:0, suggesting that the treatment may have increased the degree of saturation of adipocyte plasma membrane lipids.

The selective sorting of SFAs into EVs by SGBS adipocytes in response to PA treatment may represent a potential mechanism by which adipocytes inform other cells, in a paracrine or endocrine fashion, about their metabolic status. However, the systemic effects of these EVs on other cells and tissues, such as immune cells, liver, and skeletal muscle, warrant further investigation.

Nevertheless, while this adaptive response contributed to preventing the metabolically detrimental effects of PA on SGBS adipocytes, the increase in SFA export within EVs may represent an early stress response of adipocytes that precedes the onset of their dysfunction but may still putatively impact systemic metabolic health. Indeed, the excess SFA secreted as part of EVs may exert metabolically detrimental effects in other tissues, including the skeletal muscle and the liver. In line with this, EVs derived from hypertrophic and insulin-resistant adipocytes have been reported to be able to disrupt insulin signaling in healthy adipocytes (59). However, it remains to be determined whether EVs secreted from adipocytes with preserved insulin signaling are also able to hamper insulin sensitivity in recipient cells.

To conclude, SGBS adipocytes mount a metabolic adaptive response to rewire lipid metabolism, thereby preventing the metabolic detrimental effects of PA, at least when the treatment with this fatty acid did not exceed 48 h. These responses to PA overload also encompass the increased secretion of SFA as part of EVs, which appears to be disentangled from overt adipocyte dysfunction. Thus, enhanced packing of SFA within EVs in response to PA, while representing an additional mechanism by which adipocytes inform other cells of their metabolic status, may negatively affect systemic metabolic health even in the absence of manifest adipocyte metabolic impairments. However, it must be acknowledged that, despite SGBS adipocytes representing a physiologically relevant adipocyte model (41), these findings are specific to these cells and remain to be confirmed in other cell models. Moreover, these cells are still derived from an individual with the SGBS syndrome, and the genetic variants underpinning the phenotype of the donor remain to be fully elucidated. Additionally, it should be taken into consideration that SGBS cells are derived from the subcutaneous adipose tissue, whose role in mediating obesity comorbidities differs significantly from that played by the visceral adipose tissue (71). Finally, another important aspect to consider, beyond the intrinsic characteristics of this cell model, is that the results reported herein only apply to PA. Indeed, it remains to be elucidated whether the effects elicited by PA on adipocyte and EV lipidome are specific to this or can be promoted by other saturated fatty acids.

These findings underscore the importance of considering cell-specific responses to nutrient challenges when investigating the metabolic effects of SFAs. Future research should focus on the inter-organ and cell communication mediated by adipocyte-derived EVs and their role in systemic metabolic regulation. Moreover, our results suggest that targeting the pathways involved in EV lipid sorting and secretion could represent a novel therapeutic strategy to mitigate the adverse metabolic effects of SFA overload, particularly in the context of obesity and related metabolic disorders. However, this remains a hypothesis, and further studies are warranted to confirm the pathophysiological relevance of SFAs conveyed by EVs to metabolically active tissues such as the liver and the skeletal muscle.

## Data Availability

The original contributions presented in the study are included in the article/**Supplementary Material**. Further inquiries can be directed to the corresponding author.

## Supplementary material

Supplementary Figure 1 **Palmitic acid (PA) cytotoxicity assay on SGBS adipocytes. (**A) Cytotoxic effect of 48h treatment with 500-1000 μM of PA was evaluated on SGBS adipocytes using the LDH cytotoxicity assay. Data are expressed as percentage of LDH activity compared to the positive control obtained using cell lysate. Data are reported as mean ± SEM of four independent wells.

Supplementary Figure 2 Charter control of (A) undecanoic (C11:0) acid and (B) tricosanoate methyl ester (C23:0) internal standards (n = 12).
