# Supplementary figures and images for: Palmitic acid differently modulates extracellular vesicles and cellular fatty acid composition of SGBS adipocytes without impairing their insulin signaling
Source: Front Endocrinol (Lausanne). 2025 Nov 25;16:1699831. doi: 10.3389/fendo.2025.1699831 (PMC12685657; doi:10.3389/fendo.2025.1699831)

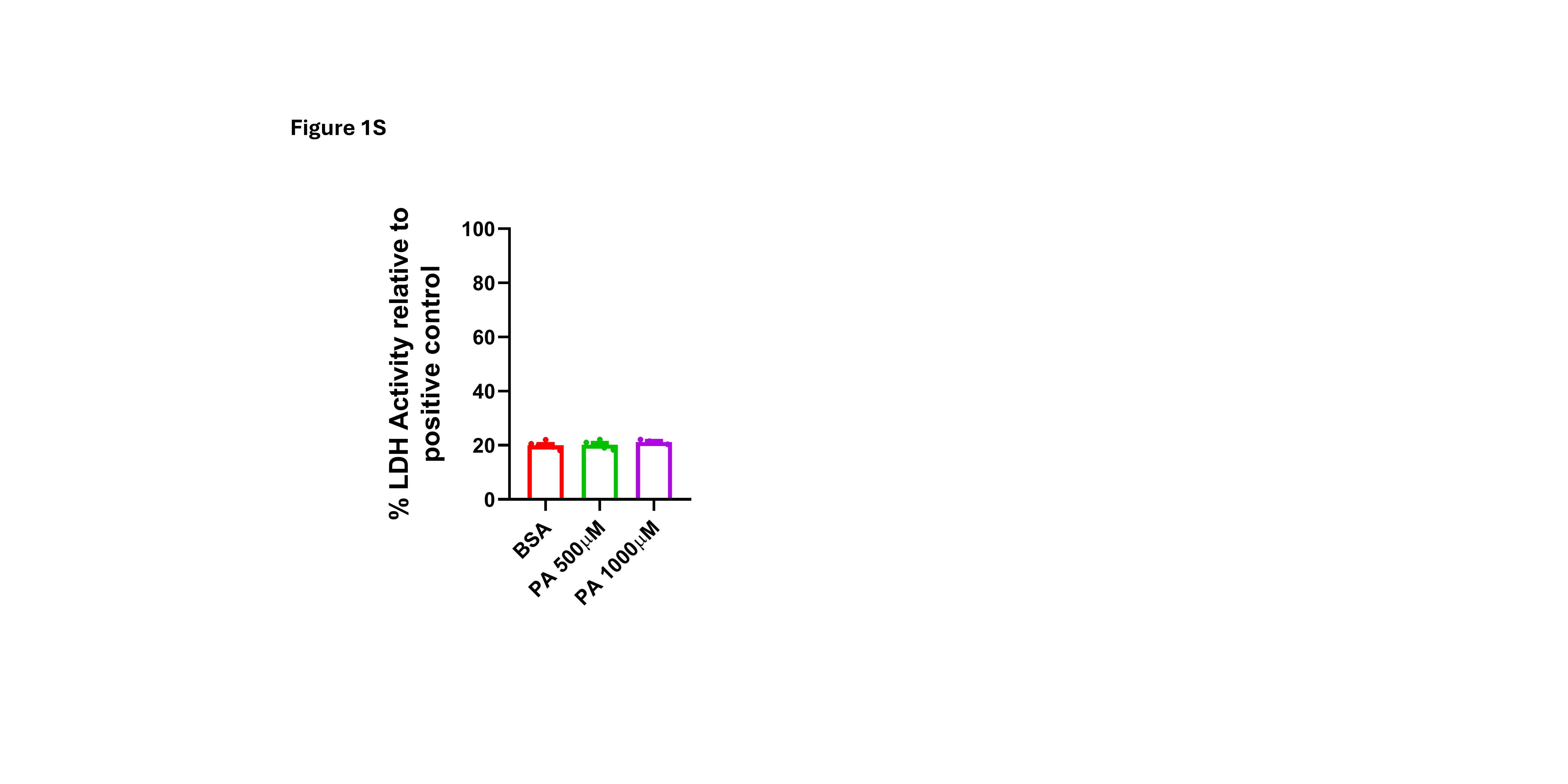

Supplement: Supplementary Figure 1 — Palmitic acid (PA) cytotoxicity assay on SGBS adipocytes. (A) Cytotoxic effect of 48h treatment with 500-1000 μM of PA was evaluated on SGBS adipocytes using the LDH cytotoxicity assay. Data are expressed as percentage of LDH activity compared to the positive control obtained using cell lysate. Data are reported as mean ± SEM of four independent wells. [file Image1.jpeg]

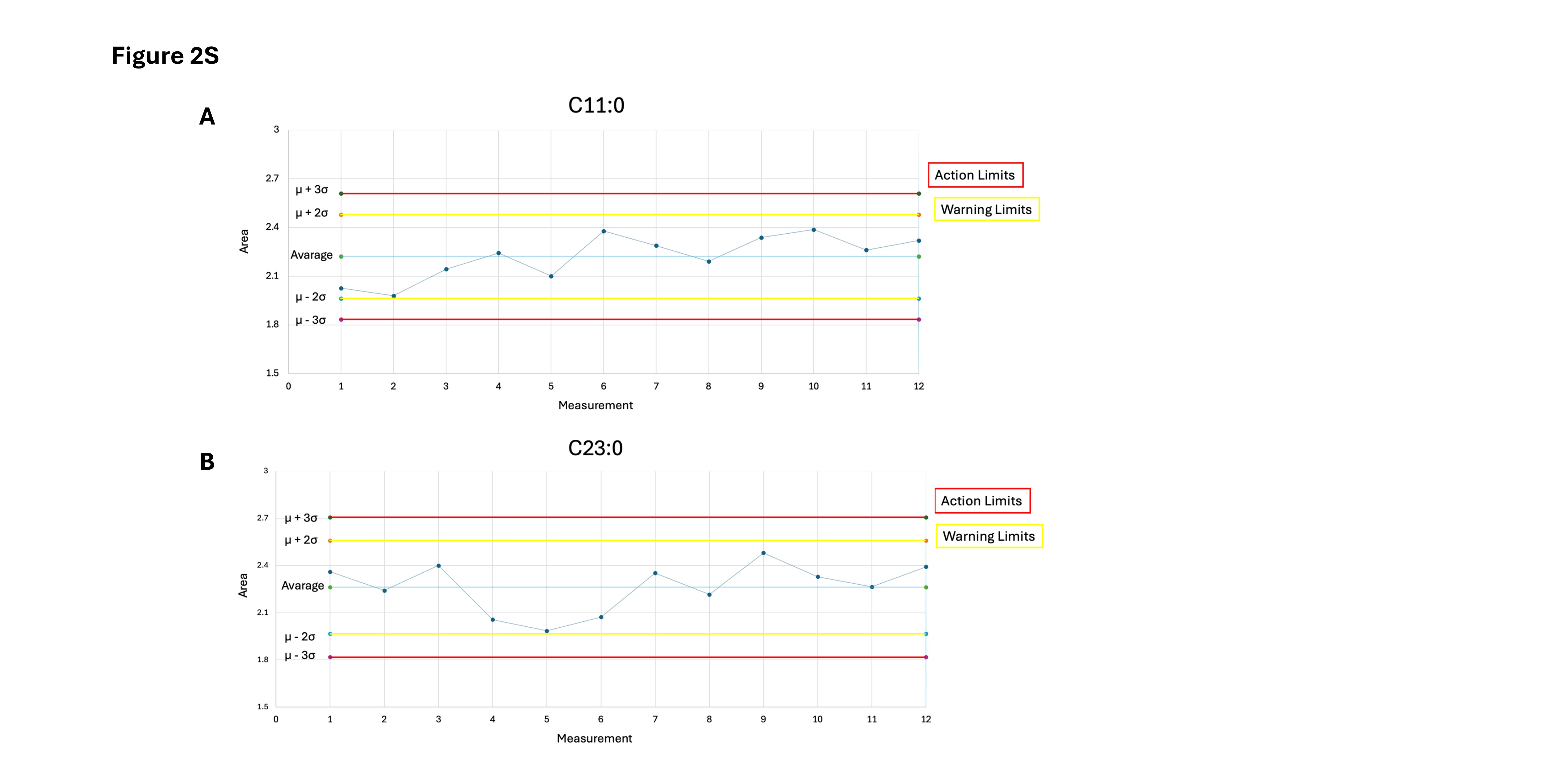

Supplement: Supplementary Figure 2 — Charter control of (A) undecanoic (C11:0) acid and (B) tricosanoate methyl ester (C23:0) internal standards (n = 12). [file Image2.jpeg]

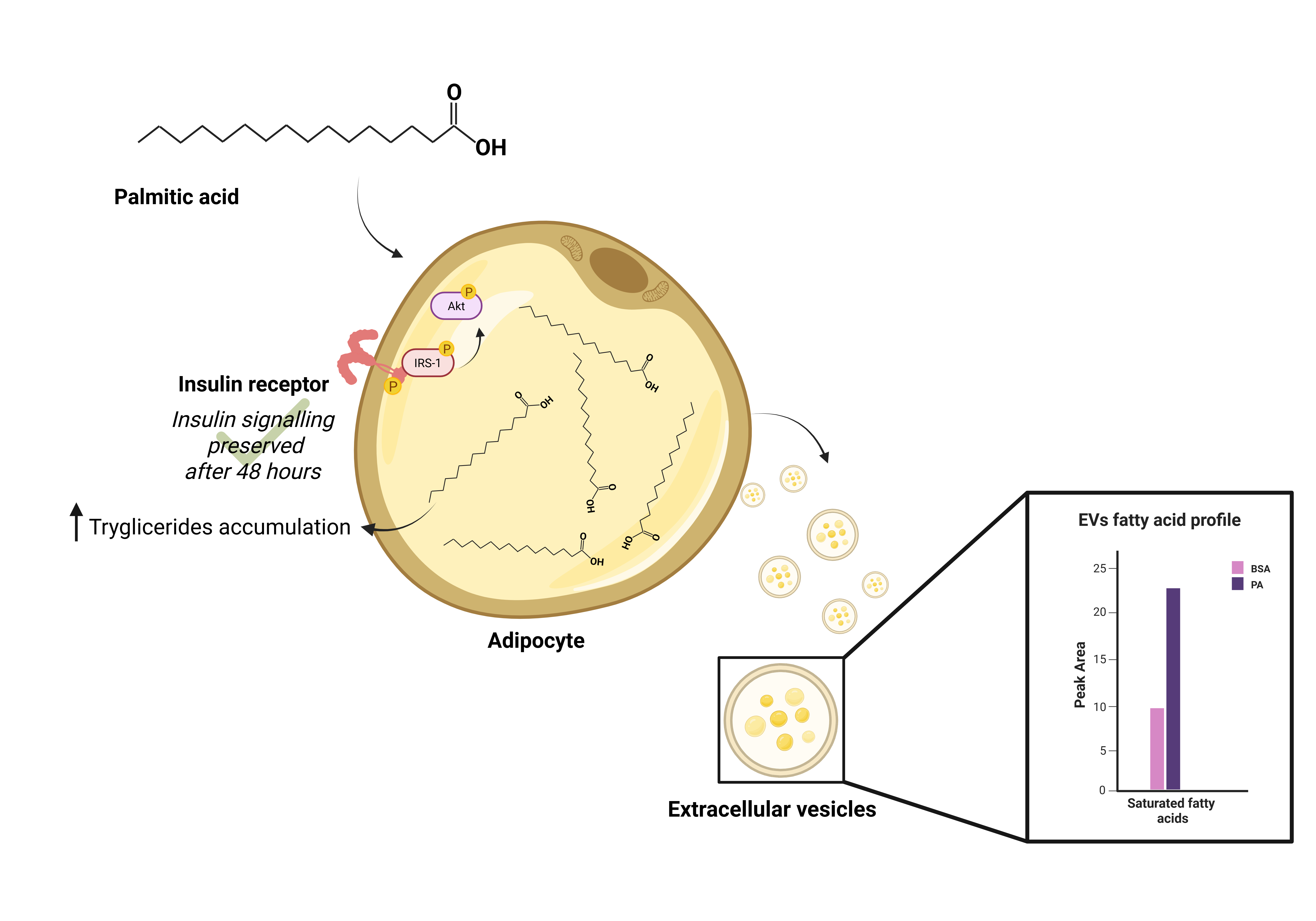

Supplement: Supplementary file 3 [file Image3.png]
